# Vitamin B6 Deficiency May Not Always Present As Microcytic Hypochromic Anemia

**DOI:** 10.7759/cureus.86768

**Published:** 2025-06-25

**Authors:** Sanshiro Nakao, Chiaki Nakaseko, Takahiro Ishii, Kensuke Terai, Naomi Shimizu

**Affiliations:** 1 Hematology, Toho University Medical Center Sakura Hospital, Sakura, JPN; 2 Hematology, International University of Health and Welfare School of Medicine, Narita, JPN; 3 Clinical Laboratory and Experimental Research Medicine, Toho University Medical Center Sakura Hospital, Sakura, JPN

**Keywords:** mean corpuscular hemoglobin, mean corpuscular hemoglobin concentration, mean corpuscular volume, myelodysplastic syndrome, vitamin b6 deficiency

## Abstract

Objective

Vitamin B6 (VB6) deficiency leads to microcytic hypochromic anemia with ringed sideroblasts. Sideroblastic anemia due to VB6 deficiency is an important differential diagnosis to consider in cases with sideroblastic anemia associated with myelodysplastic syndrome (MDS). However, VB6 screening is underutilized in Japan as it is not covered by the national health insurance system, potentially leading to low screening rates.

Methods

This study retrospectively evaluated the relationships between VB6 (pyridoxal (PAL)) and other clinical parameters, including anemia, with univariate and multivariate regression analysis. Among 43 patients, we excluded one case where vitamin B6 supplementation had already started before blood sampling. We enrolled 42 patients who had undergone measurement of VB6 level (PAL) at Toho University Sakura Medical Center (Sakura, JPN) between September 2010 and October 2023.

Results

The PAL levels were significantly correlated with mean corpuscular volume (MCV) and mean corpuscular hemoglobin (MCH). Mean corpuscular hemoglobin concentration (MCHC) and vitamin B12 (VB12) also tended to correlate positively with PAL; however, no correlation was observed between PAL levels and other parameters. The PAL was positively correlated only with MCV and MCH in the subgroup analysis of hematological disorders alone. Only alkaline phosphatase (ALP) showed a significant decrease in the PAL <6 ng/mL group compared with the PAL ≥6 ng/mL groups; no significant differences were observed in RBCs, hemoglobin (Hgb), MCV, MCH, or MCHC between the PAL <6 and ≥6 ng/mL groups in both the overall cohort of 42 patients and the subset of 12 patients with hematological disease. Multivariate regression analysis revealed that only RBC and MCV levels independently and significantly affected Hgb levels.

Conclusions

These findings suggest that anemia due to VB6 deficiency may not always present as microcytic hypochromic anemia. Thus, VB6 deficiency must be included in the differential diagnosis of anemia, especially when there's suspicion for MDS.

## Introduction

Vitamin B6 (VB6) is a vital water-soluble vitamin that plays a crucial role in heme biosynthesis by acting as a coenzyme for δ-aminolevulinic acid synthase (ALAS), the first enzyme in the heme production pathway. Its deficiency can lead to impaired hemoglobin (Hgb) synthesis, resulting in microcytic hypochromic anemia [1-3]. Vitamin B6 exists in several forms, including pyridoxine (PIN), pyridoxal (PAL), and pyridoxamine (PAM), which are converted into the active coenzyme form pyridoxal 5‘-phosphate in the body [4]. The normal ranges of PIN, PAL, and PAM are ≤3.0, 6.0-19.0, and ≤0.6 ng/mL, respectively. Among these forms, PAL plays a crucial role in various physiological functions, particularly heme synthesis, as it supports the activity of ALAS, the first enzyme in the heme biosynthesis pathway, thereby promoting RBC formation [5].

We encountered a patient with ringed sideroblasts due to VB6 deficiency anemia, which developed as a complication of the use of antidepressants. The patient also presented with microcytic hypochromic anemia, and it was difficult to differentiate from myelodysplastic syndrome (MDS). Despite its clinical significance, VB6 screening is underutilized in Japan because it is not covered by the national health insurance system, resulting in low screening rates. This study aimed to evaluate the association between VB6 deficiency and microcytic hypochromic anemia and to explore potential indicators of VB6 deficiency with general blood test parameters, as well as liver function tests.

## Materials and methods

Inclusion and exclusion criteria

A total of 43 patients who had undergone measurement of their PAL levels at Toho University Sakura Medical Center (Sakura, JPN), from September 2010 to October 2023, were retrospectively enrolled in this study, regardless of the clinical department at which they consulted. The cases in which vitamin B6 supplementation had already started before blood sampling were excluded. We analyzed only the results of the first blood sampling.

Statistical analysis

All statistical analyses were conducted using JMP software version 9.0 (SAS Institute, Cary, NC, USA). Continuous variables were assessed for normality using the Shapiro-Wilk test. Since most variables were not normally distributed, they were presented as median values with interquartile ranges (IQR).To evaluate associations between PAL (pyridoxal) levels and other clinical-laboratory parameters (including WBCs, RBCs, Hgb, mean corpuscular volume (MCV), mean corpuscular hemoglobin (MCH), MCH concentration (MCHC), platelets (PLT), reticulocytes (RET), ferritin (FER), glutamic oxaloacetic transaminase (GOT), glutamic pyruvic transaminase (GPT), alkaline phosphatase (ALP), gamma-glutamyl transferase (γ-GT), lactate dehydrogenase (LDH), total bilirubin (T-BIL), creatinine (Cre]), blood urea nitrogen (BUN), vitamin B12 (VB12]), folic acid (FA), ferrum (Fe), unsaturated iron binding capacity (UIBC), and total iron binding capacity (TIBC)), Spearman’s rank correlation coefficient was used as a non-parametric measure of statistical dependence. Correlation coefficients (ρ) and corresponding p-values were calculated for each parameter.

We used a PAL level of <6 ng/mL to predict microcytic anemia (defined as MCV < 80 fL). This cut-off demonstrated a sensitivity of 100%, a relatively high specificity of 71.8%, and a Youden index of 0.718. Based on these results, we established 6 ng/mL as the cut-off value for PAL. For group comparisons, we stratified patients based on their PAL levels (<6 ng/mL vs. ≥6 ng/mL), which is the clinical cut-off for VB6 deficiency. The Wilcoxon rank-sum test was employed to evaluate differences in continuous variables between the two groups, as the data did not follow a normal distribution. Additionally, for exploratory subgroup analyses limited to patients with hematological disorders (n=12), the same correlation and comparison methods (Spearman’s and Wilcoxon rank-sum tests) were applied. Furthermore, we used multivariate regression analysis. A two-sided p-value <0.05 was considered statistically significant for all tests.

Ethical considerations

The protocol of this study was approved by the Ethics Committee of Toho University Medical Center Sakura Hospital (approval no. S24002). The study was conducted from September 2023 to October 2024 per the Declaration of Helsinki (1975, revised in 2013). We accessed the database from September 2010 to October 2023. Our ethics committee approved an opt-out consent protocol since we used previously obtained data.

## Results

Patient characteristics and clinical parameters

Of the 43 patients, one had already been administered VB6 supplementation and was thus excluded. The characteristics and clinical parameters of the remaining 42 patients (28 males and 14 females), including 12 patients with hematological diseases, are listed in Table 1.

**Table 1:** Patient characteristics and clinical parameters PAL: Pyridoxal; Hgb: Hemoglobin; MCV: Mean corpuscular volume; MCH: Mean corpuscular hemoglobin; MCHC: Mean corpuscular hemoglobin concentration; PLT: Platelets; RET: Reticulocyte; FER: Ferritin; GOT: Glutamic oxaloacetic transaminase; GPT: Glutamic pyruvic transaminase; ALP: Alkaline phosphatase; g-GT: Gamma-glutamyl transferase; LDH: Lactate dehydrogenase; T-BIL: Total bilirubin; Cre: Creatinine; BUN: Blood urea nitrogen; VB12: Vitamin B12; FA: Folic acid; Fe: Ferrum; TIBC: Total iron binding capacity; UIBC: Unsaturated iron binding capacity; IQR: Interquartile range

Variables (unit)	Normal range	Total no. of patients		Hematological patients	
		n	Median (IQR)	n	Median (IQR)
Age (years)		42	68.5 (52.0-80.0)	12	70.5 (60.3-81.0)
Gender (m/f)		42	28/14	12	7/5
PAL (ng/mL)	6.0-19.0	42	3.9 (2.0-9.1)	12	2.0 (2.0-6.4)
WBC (/μL)	3300-9000	42	4,610 (4,003-7,155)	12	4,890 (3,945-7,663)
RBC (x10^4^/μL)	430-570	42	340 (260-400)	12	242 (214-290)
Hgb (g/dL)	13.5-17.5	42	10.6 (7.5-12.7)	12	7.2 (6.6-7.9)
MCV (fl)	85-102	42	96.0 (90.0-101.0)	12	96.5 (85.4-103.3)
MCH (pg)	28.0-34.0	42	31.9 (29.8-33.3)	12	32.3 (27.8/33.4)
MCHC (%)	30.2-35.1	42	33.3 (32.8-33.8)	12	33.2 (31.7-33.7)
PLT (x10^4^/μL)	14-34	42	22.7 (12.9-26.3)	12	17.4 (7.4-28.5)
RET (‰)	4.0-19.0	18	23 (11-28)	11	15 (9-26)
FER (ng/mL)	21.81-274.66	17	348 (101-740)	9	348 (101-639)
GOT (IU/L)	10-40	40	27 (19-40)	12	24 (16-28)
GPT (IU/L)	5-45	40	20 (11-43)	12	13 (11-66)
ALP (IU/L)	38-113	39	202 (122-288)	12	118 (64-216)
γ-GT (IU/L)	<80	32	41 (24-76)	12	37 (15-81)
LDH (IU/L)	124-222	40	201 (165-254)	12	197 (164-227)
T-BIL (mg/dL)	0.2-1.2	41	0.9 (0.6-1.1)	12	0.8 (0.7-0.9)
Cre (mg/dL)	0.61-1.04	41	0.8 (0.6-0.9)	12	0.8 (0.7-0.9)
BUN (mg/dL)	8.0-20.0	41	16.2 (11.1-20.3)	12	13.7 (9.6-21.3)
VB12 (pg/mL)	233-914	27	572 (399-1195)	4	886.5 (427.5-1372.5)
FA (ng/mL)	3.6-12.9	20	8.6 (5.2-11.1)	4	7.3 (5.7-10.9)
Fe (μg/dL)	50-200	15	92.0 (49.5-141.0)	6	164.5 (121.8-174.3)
TIBC (μg/dL)	270-425	14	244.0 (199.3-281.3)	6	225.0 (185.5-265.3)
UIBC (μg/dL)	140-330	14	154.0 (78.3-246.8)	6	87.0 (33.3-224.0)

The median age of the patients was 68.5 years (IQR: 52.0-80.0). Their PAL levels ranged from 2.0 to 9.1 ng/mL (median: 3.9 ng/mL). All samples were within the reference range of Wakosil-Ⅱ 5C18HG. The distribution of VB6 submissions was as follows: 12 cases from the Department of Hematology, six from the Department of Neurology, five each from the Departments of Metabolism and Respiratory Medicine, four each from the Departments of Cardiology and Gastroenterology, and six from other departments. In hematology, VB6 was primarily measured to investigate the cause of anemia. In psychiatry, it was assessed due to its potential involvement in the development of depression. Additionally, several medications commonly prescribed in psychiatric practice-such as isoniazid, carbamazepine, phenytoin, and certain antidepressants, can interfere with VB6 metabolism, prompting further evaluation. Beyond these reasons, VB6 testing was also requested for the assessment of numbness or tingling symptoms, alcohol-related neurological conditions, and cases with suspected poor nutritional status. The breakdown of hematological disorders was as follows: seven cases of MDS, and one case each of acute myeloid leukemia (AML), malignant lymphoma, zinc deficiency anemia, VB6 deficiency anemia, and pure red cell aplasia (PRCA). The median Hgb level was 10.6 g/dL in 42 patients and 7.2 g/dL in patients with hematological diseases. The median MCV, MCH, and MCHC values were almost the same in the two groups.

Correlation analysis between PAL and clinical variables

The correlation between PAL levels and various clinical parameters was determined using Spearman’s rank correlation analysis. The PAL levels were significantly and positively correlated with MCV (ρ = 0.3725, p = 0.0151) and MCH (ρ = 0.4047, p = 0.0079) (Table 2). Moreover, a weak but not statistically significant positive correlation was observed between PAL and both VB12 (p = 0.0582) and MCHC (p = 0.0732). However, no correlation was observed between PAL and other parameters, including Hgb, Fe, TIBC, and UIBC. 

**Table 2:** Correlation analysis between PAL and clinical variables Statistical analysis was performed using Spearman’s rank correlation analysis. *A p-value < 0.05 is considered statistically significant. PAL: Pyridoxal; Hgb: Hemoglobin; MCV: Mean corpuscular volume; MCH: Mean corpuscular hemoglobin; MCHC: Mean corpuscular hemoglobin concentration; PLT: Platelets; RET: Reticulocyte; FER: Ferritin; GOT: Glutamic oxaloacetic transaminase; GPT: Glutamic pyruvic transaminase; ALP: Alkaline phosphatase; g-GT: Gamma-glutamyl transferase; LDH: Lactate dehydrogenase; T-BIL: Total bilirubin; Cre: Creatinine; BUN: Blood urea nitrogen; VB12: Vitamin B12; FA: Folic acid; Fe: Ferrum; TIBC: Total iron binding capacity; UIBC: Unsaturated iron binding capacity

Variable (n)	Spearman's coefficient (ρ)	p-value
WBC (42)	-0.0129	0.9356
RBC (42)	-0.1327	0.4022
Hgb (42)	0.0138	0.9307
MCV (42)	0.3725	0.0151*
MCH (42)	0.4047	0.0079*
MCHC (42)	0.2793	0.0732
PLT (42)	0.0409	0.7969
RET (18)	0.0054	0.9831
FER (17)	-0.153	0.5578
GOT (40)	0.0402	0.8056
GPT (40)	-0.0641	0.6945
ALP (39)	-0.0394	0.812
γ-GT (32)	0.0429	0.8157
LDH (40)	0.0287	0.8605
T-BIL (41)	-0.184	0.2495
Cre (41)	0.1415	0.3776
BUN (41)	0.053	0.7419
VB12 (27)	0.369	0.0582
FA(20)	-0.2445	0.2988
Fe (15)	-0.1019	0.7177
TIBC (14)	0.0007	0.9981
UIBC (14)	-0.0529	0.8574

To minimize the impact of confounding factors arising from the inclusion of heterogeneous diseases, we additionally performed an analysis only on cases that had undergone comprehensive evaluation at the Department of Hematology. As shown in Table 3, a strong positive correlation was observed between PAL and MCV (ρ = 0.6803, p = 0.0149) and MCH (ρ = 0.6637, p = 0.0186). While no correlation was identified between PAL and Hgb, a negative correlation was observed between PAL and RBC (ρ = −0.6256, p = 0.0296). Folic acid and VB12 levels were measured in only four cases, and Fe, TIBC, and UIBC levels in only six cases; therefore, we were unable to perform statistical analyses on these variables. 

**Table 3:** Correlation analysis between PAL and clinical variables of the patients evaluated in the Department of Hematology Statistical analysis was performed using Spearman’s rank correlation analysis. *A p-value < 0.05 is considered statistically significant. PAL: Pyridoxal; Hgb: Hemoglobin; MCV: Mean corpuscular volume; MCH: Mean corpuscular hemoglobin; MCHC: Mean corpuscular hemoglobin concentration; PLT: Platelets; RET: Reticulocyte; FER: Ferritin; GOT: Glutamic oxaloacetic transaminase; GPT: Glutamic pyruvic transaminase; ALP: Alkaline phosphatase; g-GT: Gamma-glutamyl transferase; LDH: Lactate dehydrogenase; T-BIL: Total bilirubin; Cre: Creatinine; BUN: Blood urea nitrogen; VB12: Vitamin B12; FA: Folic acid; Fe: Ferrum; TIBC: Total iron binding capacity; UIBC: Unsaturated iron binding capacity

Variable (n)	Spearman's coefficient (ρ)	p-value
WBC (12)	0.5918	0.0427
RBC (12)	-0.6256	0.0296*
Hgb (12)	-0.2632	0.4085
MCV (12)	0.6803	0.0149*
MCH (12)	0.6637	0.0186*
MCHC (12)	0.3992	0.1985
PLT (12)	-0.2374	0.4576
RET (11)	0.1206	0.724
FER (9)	0.1825	0.6383
GOT (12)	0.0702	0.8284
GPT (12)	0.0324	0.9203
ALP (12)	0.2866	0.3665
γ-GT (12)	0.5976	0.0402*
LDH (12)	0.4116	0.1837
T-BIL (12)	-0.1627	0.6134
Cre (12)	-0.0867	0.7888
BUN (12)	-0.1676	0.6027

Comparison of clinical parameters between the PAL groups

Next, we compared several clinical parameters between the PAL <6 and ≥6 ng/mL groups using Wilcoxon analysis. As shown in Table 4, no significant differences in RBC, Hgb, VB12, and FA levels were observed between the two groups. However, these values tended to be lower in the PAL < 6 ng/mL group. Meanwhile, MCV, MCH, and MCHC did not show low values, even in the PAL <6 ng/mL group. 

**Table 4:** Comparison of clinical parameters between the PAL <6 ng/mL and PAL ≥ 6 ng/mL groups Statistical analysis was performed using the Wilcoxon rank-sum test. *A p-value < 0.05 is considered statistically significant. PAL: Pyridoxal; Hgb: Hemoglobin; MCV: Mean corpuscular volume; MCH: Mean corpuscular hemoglobin; MCHC: Mean corpuscular hemoglobin concentration; PLT: Platelets; RET: Reticulocyte; FER: Ferritin; GOT: Glutamic oxaloacetic transaminase; GPT: Glutamic pyruvic transaminase; ALP: Alkaline phosphatase; g-GT: Gamma-glutamyl transferase; LDH: Lactate dehydrogenase; T-BIL: Total bilirubin; Cre: Creatinine; BUN: Blood urea nitrogen; VB12: Vitamin B12; FA: Folic acid; Fe: Ferrum; TIBC: Total iron binding capacity; UIBC: Unsaturated iron binding capacity

Variable (unit)	n	PAL < 6			PAL ≥ 6			p-value
		Median	n	Range	Median	n	Range	
WBC (/μL)	42	4450	14	2000-9670	4740	28	3060-12400	0.4312
RBC (x10^4^/μL)	42	305	14	114-596	373	28	146-544	0.2681
Hgb (g/dL)	42	8.5	14	4.7-18.0	11.2	28	6.0-15.4	0.1386
MCV (fl)	42	97.5	14	68.0-124.0	95.5	28	82.0-124.0	0.7996
MCH (pg)	42	32.8	14	19.9-41.2	31.8	28	25.3-42.8	0.7999
MCHC (%)	42	33.2	14	28.9-33.9	33.4	28	30.8-35.1	0.1952
PLT (x10^4^/μL)	42	20.2	14	3.1-62.3	22.7	28	2.9-70.7	0.6598
RET (‰)	18	18	8	4-45	25	10	6-102	0.3986
FER (ng/mL)	17	739.8	7	43.6-8334.6	263.4	10	6.2-1038.2	0.0877
GOT (IU/L)	40	25	13	14-43	27	27	12-109	0.3331
GPT (IU/L)	40	11	13	5-63	20	27	7-120	0.1742
ALP (IU/L)	39	129	13	38-605	209	22	67-885	0.0398*
γ-GT (IU/L)	32	47	10	12-244	40	22	13-550	0.6841
LDH (IU/L)	40	191	14	102-489	203	26	124-349	0.8537
T-BIL (mg/dL)	41	1.0	14	0.2-1.8	0.7	27	0.5-2.0	0.2237
Cre (mg/dL)	41	0.84	14	0.3-3.2	0.72	27	0.4-1.7	0.2835
BUN (mg/dL)	41	18.2	14	5.9-112.5	13.5	27	5.8-34.5	0.134
Vit B12 (pg/mL)	27	495	9	313-1500	691	18	255-1500	0.7374
Folic acid (ng/mL)	20	7.3	8	4.8-20.0	10.5	12	2.9-20.0	0.6432
Fe (μg/dL)	15	110	5	11.0-190.0	78.0	10	25.0-175.0	0.7595
TIBC (μg/dL)	14	149.5	4	15.0-251.0	270	10	181.0-505.0	0.0560
UIBC (μg/dL)	14	81.5	4	61.0-261.0	194.5	10	9.0-480.0	0.4367

We also performed the same analyses between the PAL < 6 ng/mL and PAL ≥ 6 ng/mL groups in only patients with hematological disorders. In the PAL < 6 ng/mL group (n = 6) and PAL ≥ 6 ng/mL group (n = 6), the median and range of each variable in those with anemia are shown in Table 5. The PAL < 6 ng/mL group showed a tendency toward microcytic hypochromic anemia, but the difference between the two groups did not reach statistical significance. Only ALP was significantly higher in the PAL ≥ 6 ng/mL group than in the PAL < 6 ng/mL group (p = 0.0202 by Wilcoxon rank-sum test). 

**Table 5:** Comparison of clinical parameters between the PAL <6 ng/mL and PAL ≥ 6 ng/mL groups evaluated in the Department of Hematology Statistical analysis was performed using the Wilcoxon rank-sum test. *A p-value < 0.05 is considered statistically significant. PAL: Pyridoxal; Hgb: Hemoglobin; MCV: Mean corpuscular volume; MCH: Mean corpuscular hemoglobin; MCHC: Mean corpuscular hemoglobin concentration; PLT: Platelets; RET: Reticulocyte; FER: Ferritin; GOT: Glutamic oxaloacetic transaminase; GPT: Glutamic pyruvic transaminase; ALP: Alkaline phosphatase; g-GT: Gamma-glutamyl transferase; LDH: Lactate dehydrogenase; T-BIL: Total bilirubin; Cre: Creatinine; BUN: Blood urea nitrogen

Variable (unit)	n	PAL < 6			PAL ≥ 6			p-value
		Median	n	Range	Median	n	Range	
WBC (/μL)	12	4575	6	3580-6780	7650	6	3200-12400	0.3367
RBC (x10^4^/μL)	12	290	6	170-356	226	6	146-291	0.2002
Hgb (g/dL)	12	7.0	6	5.6-9.5	7.5	6	6.0-9.4	0.6304
MCV (fl)	12	85.8	6	68.0-104.0	99.5	6	89.0-124.0	0.2002
MCH (pg)	12	28.0	6	19.9-32.9	33.3	6	29.2-41.8	0.1488
MCHC (%)	12	31.7	6	28.9-33.8	33.7	6	32.7-35.1	0.0547
PLT (x10^4^/μL)	12	19.7	6	4.4-62.3	17.4	6	2.9-27.5	0.6310
RET (‰)	11	12	6	4-45	25	5	6-31	0.3613
FER (ng/mL)	9	366.2	4	43.6-1098.0	348.0	5	6.2-1038.2	0.8065
GOT (IU/L)	12	21	6	14-31	25	6	16-109	0.2281
GPT (IU/L)	12	11	6	10-63	45	6	8-120	0.5189
ALP (IU/L)	12	62	6	38-129	227	6	67-885	0.0202*
γ-GT (IU/L)	12	27	6	12-117	37	6	16-128	0.4225
LDH (IU/L)	12	178	6	119-238	215	6	160-270	0.2623
T-BIL (mg/dL)	12	1.0	6	0.2-1.8	0.8	6	0.6-0.9	0.3333
Cre (mg/dL)	12	0.75	6	0.33-3.15	0.82	6	0.61-1.51	0.6310
BUN (mg/dL)	12	14.6	6	5.9-112.5	13.5	6	7.7-34.5	0.8728

Multivariate regression analysis of correlation with Hgb

Table 6 presents the results of the multivariate regression analysis for the correlation of Hgb with other clinical parameters. We observed that RBC level (β-coefficient = 1.201, p < 0.001) and MCV level (β-coefficient = 0.526, p < 0.001) were independent predictors of the Hgb levels. However, the PAL level was not identified as a contributing factor to Hgb levels. 

**Table 6:** Multivariate regression analysis of correlation with Hgb in all patients Model: r2 = 0.97, p < 0.0001 SE: Standard error; VIF: Variance inflation factor; Hgb: Hemoglobin; PAL: Pyridoxal; MCV: Mean corpuscular volume; ALP: Alkaline phosphatase; VB12: Vitamin B12

Variable	β-coefficient	SE	p-value	VIF
PAL	0.026	0.001	0.618	1.354
RBC	1.201	0.002	<0.001	1.689
MCV	0.526	0.012	<0.001	1.888
ALP	0.043	0.001	0.356	1.093
VB12	0.026	0.000	0.600	1.268

## Discussion

In this study, the relationship between VB6 levels and other clinical parameters, including anemia, was investigated in 42 patients. This study included one case diagnosed with MDS with ringed sideroblasts, presenting with microcytic hypochromic anemia; this was subsequently shown to be caused by VB6 deficiency resulting from impaired absorption associated with the use of an antidepressant. Following VB6 supplementation, this patient was no longer dependent on transfusion.

Previous case reports of VB6 deficiency anemia have described it as microcytic hypochromic anemia [2,3]. A positive correlation was found between PAL and MCV, as well as between PAL and MCH (Tables 2-3). However, a decrease in PAL does not necessarily result in microcytic hypochromic anemia (Tables 4-5).

Sideroblastic anemia with VB6 deficiency is an important differential diagnosis in sideroblastic anemia associated with MDS [6]. Hematological findings in patients with VB6-deficient anemia often include low Hgb levels and low MCV, with advanced-stage patients showing a hallmark feature of ringed sideroblasts in the bone marrow. Clinically, patients may experience fatigue, weakness, and pallor, which are common symptoms of anemia. Microcytic hypochromic anemia was neither observed in the PAL <6 ng/mL group (Table 4) nor in the analysis of those with hematological diseases (Table 5). Furthermore, the PAL level was not identified as a contributing factor to Hgb levels by multivariate regression analysis (Table 6).

Given the weak positive correlation between PAL and VB12 (Table 2), it was suggested that, in conjunction with megaloblastic anemia caused by VB12 and FA deficiency, VB6 deficiency anemia may not always present as microcytic hypochromic anemia. Vitamin B6 deficiency has been reported to be associated with deficiencies of other vitamins and has been shown to frequently coexist with vitamin D deficiency [7]. Furthermore, concurrent deficiencies of VB6 and FA have been reported to be associated with an increased risk of depressive symptoms [8].

Vitamin B6 acts as a coenzyme in the amino group transfer reactions of GOT and GPT [9]. In particular, VB6 plays an essential role when these enzymes transfer amino groups to other molecules. Upon deficiency of VB6, the activity of these enzymes may decrease, leading to impaired amino acid metabolism. Therefore, VB6 deficiency may result in reduced GOT and GPT activity [10,11]. As shown in Tables 2 and 3, no significant correlation was observed between VB6 level and GOT or GPT activity.

Alkaline phosphatase was significantly lower in the PAL <6 ng/mL group than in the PAL ≥6 ng/mL group (p = 0.0398) (Table 4), also in those with hematological diseases (p = 0.0202) (Table 5). In VB6 metabolism, dephosphorylation by ALP is required before its absorption and subsequent phosphorylation in the body. In healthy adults, common variants in ALP influence plasma pyridoxal 5’-phosphate [12]. Therefore, we hypothesized that if a decrease in ALP affects VB6 metabolism or utilization efficiency, there is a need to consider the influence of specific diseases or nutritional status [13].

To our knowledge, there have been no comprehensive reports on VB6 deficiency and anemia to date, and this study is the first to report on the subject. However, this study has several limitations. First, this study focused on a single center, small sample size, and included cases of gastrointestinal diseases, in addition to those of hematological disorders, and other diseases. Since multiple diseases were included, only the cases of hematological disorders could be included for analysis. However, the obtained results were almost identical to those from the overall analysis. Second, the data for VB12, FA, Fe, UIBC, and TIBC, aside from PAL, were also incomplete. Third, the potential confounding factors, such as nutritional status and medication use, were not controlled. To comprehensively investigate anemia caused by VB6, analyzing a larger cohort with a sole focus on hematological disorders is essential.

## Conclusions

In this study, we investigated the relationship between VB6 levels and various clinical parameters and found that VB6 deficiency does not necessarily present as microcytic hypochromic anemia. Additionally, the VB6-deficient group exhibited significantly lower ALP levels, suggesting that reduced enzymatic activity related to VB6 absorption and metabolism may be involved. These findings underscore the importance of including VB6 deficiency in the differential diagnosis of anemia and prompt reconsideration of the current underutilization of VB6 testing, especially in cases suspected of MDS. Moving forward, wider implementation of VB6 screening in patients with unexplained anemia may improve diagnostic accuracy and contribute to better treatment strategies.
